# How to “Start Small and Just Keep Moving Forward”: Mixed Methods Results From a Stepped-Wedge Trial to Support Evidence-Based Processes in Local Health Departments

**DOI:** 10.3389/fpubh.2022.853791

**Published:** 2022-04-28

**Authors:** Rebekah R. Jacob, Renee G. Parks, Peg Allen, Stephanie Mazzucca, Yan Yan, Sarah Kang, Debra Dekker, Ross C. Brownson

**Affiliations:** ^1^Prevention Research Center, Brown School, Washington University in St. Louis, St. Louis, MO, United States; ^2^Division of Public Health Sciences, Department of Surgery, Alvin J. Siteman Cancer Center, Washington University School of Medicine, Washington University in St. Louis, St. Louis, MO, United States; ^3^Fredrick S. Pardee RAND Graduate School, RAND Corporation, Santa Monica, CA, United States; ^4^National Association of County and City Health Officials, Washington, DC, United States

**Keywords:** evidence-based decision making, evidence-based public health, local health department, organizational capacity, evidence-based decision making competency

## Abstract

**Background:**

Local health departments (LHDs) in the United States are charged with preventing disease and promoting health in their respective communities. Understanding and addressing what supports LHD's need to foster a climate and culture supportive of evidence-based decision making (EBDM) processes can enhance delivery of effective practices and services.

**Methods:**

We employed a stepped-wedge trial design to test staggered delivery of implementation supports in 12 LHDs (Missouri, USA) to expand capacity for EBDM processes. The intervention was an in-person training in EBDM and continued support by the research team over 24 months (March 2018–February 2020). We used a mixed-methods approach to evaluate: (1) individuals' EBDM skills, (2) organizational supports for EBDM, and (3) administered evidence-based interventions. LHD staff completed a quantitative survey at 4 time points measuring their EBDM skills, organizational supports, and evidence-based interventions. We selected 4 LHDs with high contact and engagement during the intervention period to interview staff (n = 17) about facilitators and barriers to EBDM. We used mixed-effects linear regression to examine quantitative survey outcomes. Interviews were transcribed verbatim and coded through a dual independent process.

**Results:**

Overall, 519 LHD staff were eligible and invited to complete quantitative surveys during control periods and 593 during intervention (365 unique individuals). A total of 434 completed during control and 492 during intervention (83.6 and 83.0% response, respectively). In both trial modes, half the participants had at least a master's degree (49.7–51.7%) and most were female (82.1–83.8%). No significant intervention effects were found in EBDM skills or in implementing evidence-based interventions. Two organizational supports scores decreased in intervention vs. control periods: awareness (−0.14, 95% CI −0.26 to −0.01, *p* < 0.05) and climate cultivation (−0.14, 95% CI −0.27 to −0.02, *p* < 0.05) but improved over time among all participants. Interviewees noted staff turnover, limited time, resources and momentum as challenges to continue EBDM work. Setting expectations, programmatic reviews, and pre-existing practices were seen as facilitators.

**Conclusions:**

Challenges (e.g., turnover, resources) may disrupt LHDs' abilities to fully embed organizational processes which support EBDM. This study and related literature provides understanding on how best to support LHDs in building capacity to use and sustain evidence-based practices.

## Introduction

Local health departments (LHDs) serve as an important frontline for chronic disease prevention in the complex US public health system (1, 2). Because LHDs are more localized than state-based and national efforts, they are able to tailor the implementation of important evidence-based programs and policies to their community's needs and resources. The burden of chronic diseases like diabetes and prediabetes continues to increase, disproportionally impacting underserved communities (3). Supporting the capacity of LHDs to implement effective local strategies is an urgent priority (4). Such capacity requires skilled staff and organizational practices that support evidence-based decision making (EBDM) processes, or strategies to apply the best available scientific evidence and community preferences (5, 6). LHDs face unique challenges to building capacity, such as staff turnover, limited resources, and funding (7).

Providing training on EBDM to health department staff has been documented as an important and effective strategy to boost staff competency among the workforce and influence organizational practices (8–11). Previous work with state health departments suggests training and additional researcher-supported, agency-planned strategies to embed EBDM into systems could also enhance individual and organizational capacity to adopt evidence-based approaches (9). It is unknown if similar supports yield similar results within LHDs given the differences in governance and other organizational factors. Research to understand how best to build capacity for EBDM within LHDs has mostly been cross-sectional or longitudinal with pre-post follow-up (either with or without control groups). For the current study, we utilized a stepped-wedge design, which allows for pre-post comparisons across intervention and control groups while assuring all groups receive the possible benefits from inclusion in intervention.

In 2018, the Adoption & Implementation of evidence to Mobilize Local Health (AIM-Local Health) trial began and we recruited 12 LHDs in Missouri with the goal of understanding how training and ongoing support and technical assistance could aid LHDs and their unique context in improving capacity to use EBDM, especially with regard to chronic disease prevention. We report qualitative interview findings from LHD staff participants and quantitative survey results which are, to our knowledge, the first to feature a mixed-method, stepped-wedge cluster randomized trial with LHD as the cluster.

## Methods

This study reports results from phase 2 of a two-phase dissemination trial, grounded in Diffusion of Innovation Theory (12) and Institutional Theory (13–15), to test the effectiveness of strategies designed to increase capacity for evidence-based diabetes and other chronic disease control efforts among local public health practitioners (16). Phase 1 included a national cross-sectional survey of LHDs and qualitative interviews with key informants. Findings from Phase 1 are reported elsewhere (17–21) and were used to refine the dissemination approach and measures used in phase 2. Likewise, a full protocol for all phases has been described in detail previously (16). Here we briefly describe components related to understanding Phase 2 trial results.

### Site Selection and Study Design

**Figure 1:**
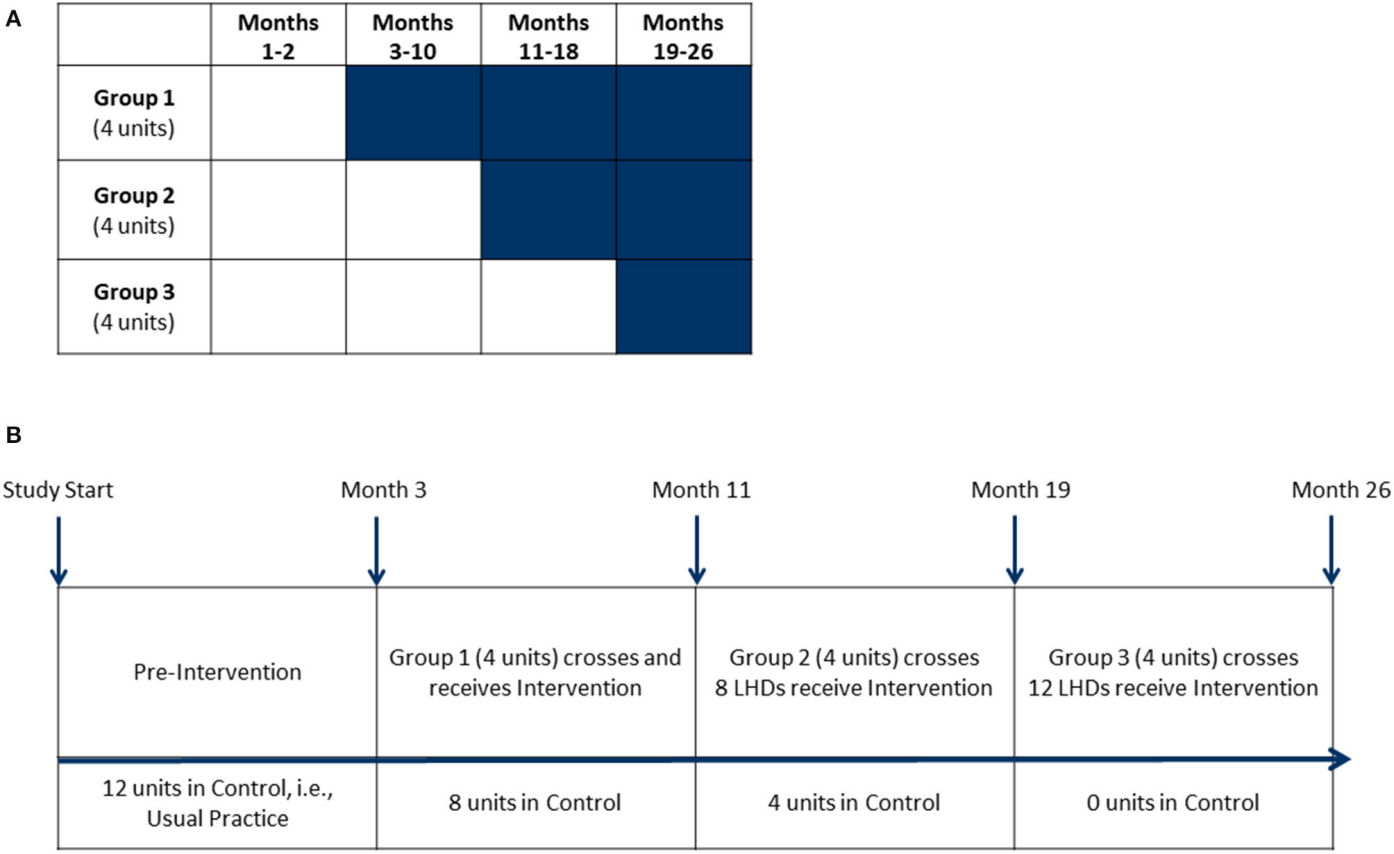
Stepped-wedge design. **(A)** This stepped-wedge design featured 12 units (local health departments) randomly assigned into one of three groups. Shaded cells represent intervention periods. Clear cells represent control periods. Group 1's intervention period was 24 months, Group 2's intervention period was 16 months and Group 3's intervention period was 8 months. **(B)** Baseline measures for all units were taken during the pre-intervention period. Groups crossed over from control to receive intervention activities with measurements at 8-month intervals.

This study used an open cohort stepped-wedge design with groups of clusters (LHDs) that crossed over to intervention at various intervals. Each cohort of participants included newly recruited and previously recruited individual participants within clusters (22, 23). In comparison to parallel trial designs, the main strength of a stepped-wedge design is all groups eventually receive the intervention. We selected 12 LHDs (from 115 LHDs) in Missouri, USA based upon several characteristics such as full-time equivalent employees (proxy for LHD size), number of people working in diabetes programs (at least 5 required), and diabetes burden-mortality rate for diabetes (disparity measure). After obtaining permission from each LHD's leadership, the LHDs were randomized into three groups (four LHDs in each group). LHDs assigned to group “1” crossed over from control into intervention first and remained in intervention status until the completion of the study (Figure 1).

### Intervention

Previous work demonstrated capacity building within health departments to be highly nuanced to each individual organization because of differing resources (21, 24). As such, the primary intervention component included a tailored evidence-based public health (EBPH) training with each LHD and continued follow-up (e.g., email exchanges, phone meetings) to determine specific areas for capacity building activities. Each LHD's team included 1–3 staff who served as key contacts with the research team. The research team included the study's principal investigator (RCB), the project manager (RGP) and expert consultant (PA).

Eight to 9 weeks after receiving the pre-intervention survey, the intervention group received a 3.5-day, in-person EBPH training led by the research team. The EBPH training was modeled from previously successful workshops to enhance EBDM within local and state health departments (25, 26). Didactic and interactive group work covers 10 modules:

1. Introduction and overview of EBPH,
2. Assessing and engaging communities,
3. Quantifying the issue,
4. Developing a concise statement of the issue,
5. Searching and summarizing the scientific literature,
6. Developing and prioritizing intervention options,
7. Developing an action plan and building a logic model,
8. Understanding and using economic evaluation,
9. Evaluating the program or policy, and
10. Communicating and disseminating evidence to local policymakers.

More information on the course and specific learning objectives for each module can be found at evidencebasedpublichealth.org and in previously published evaluation work (26, 27).

Following the training, each LHD was offered an in-person planning session with the research team to identify priority areas. LHDs chose from a list of strategies developed in previous work or proposed alternate approaches (9, 16) in the areas of accreditation, access to scientific information, workforce development, leadership and management supports, organizational changes, relationships and partnerships, and financial practices. Additional support or “check in” meetings were offered to each LHD at whatever frequency was most helpful to the LHD for their chosen strategies. See Supplementary Material 2 for additional descriptions and examples of types of supports offered to each LHD.

We tracked intervention delivery and implementation in several ways (Table 1). We logged each LHD's training start, post-training in-person planning meeting, and follow-up check-in meetings with key staff. In addition, we tracked each LHD's management strategies that were implemented during the intervention phase. All components of this study received approval by the Washington University in St. Louis Institutional Review board (#201705026 and #202010031).

**Table 1:** Intervention delivery description by group and unit (local health department).

	**LHD A**	**LHD B**	**LHD C**	**LHD D**	**LHD E**	**LHD F**	**LHD G**	**LHD H**	**LHD I**	**LHD J**	**LHD K**	**LHD L**
**Group/wave characteristics**												
Group/wave^a^	1				2				3			
Average number of full time equivalent employees (FTEs)^b^	92.0				86.5				144.1			
Average jurisdiction population (per 1,000)^c^	233.7				210.5				414.6			
Number of LHDs accredited by the Public Health Accreditation Board or Missouri Institute for Community Health at baseline	2/4				4/4				3/4			
**Intervention delivery**												
Total months in intervention phase^d^	24				16				8			
**EBPH Training**												
Month and year of training date (intervention commencement)^e^	March 2018				November 2018				July 2019			
Number of individuals trained at initial EBDM course	4	5	9	9	9	3	10	6	4	4	5	8
**Contact**												
In-person planning meeting	Yes	Yes	Yes	Yes	Yes	Yes	Yes	Yes	No	Yes	Yes	Yes
Ratio of number of meetings with research team to number of months in intervention phase	0.21 (5/24)	0.33 (8/24)	0.25 (6/24)	0.63 (15/24)	0.31 (5/16)	0.63 (5/16)	0.56 (9/16)	0.50 (8/16)	0.0 (0/8)	0.38 (3/8)	0.50 (4/8)	0.38 (3/8)
**Management Practices Implemented^f^**												
Requested (and received) TA for evaluation plan	No	No	No	Yes	Yes (x2)	No	Yes (x3)	No	Yes	Yes	No	No
Established EBPH committee	No	No	No	Yes	No	Yes	No	Yes	No	No	Yes	No
Provided additional training	No	Yes	No	Yes	Yes	Yes	No	Yes	No	Yes	No	No
Updated procedures or policies	No	No	No	Yes	No	Yes	Yes	No	No	No	Yes	No
Created process for new program selection	No	No	Yes	Yes	No	No	No	Yes	No	No	No	No
Reviewed current programs	No	Yes	No	No	No	Yes	Yes	No	No	Yes	No	No
Reviewed strategic plan or CHIP for EBPH/Use of EBIs	No	Yes	No	No	Yes	Yes	No	No	No	No	No	Yes
Additional EBPH capacity building accomplishments	No	No	No	Yes	Yes	Yes	Yes	Yes	No	No	No	No
**Data collection**												
**Quantitative survey**												
Average eligible invitees (timepoint range)	14.5 (14–16)	11.0 (9–12)	32.8 (24–39)	28.3 (25–31)	65.8 (52–81)	8.5 (8–9)	24.3 (23–25)	22.3 (21–24)	21.0 (18–24)	19.5 (19–21)	17.0 (16–18)	13.3 (12–16)
Average number of completed surveys (time point range)	12.5 (11–15)	10 (8–11)	26.3 (21–32)	26.5 (24–29)	46.8 (34–63)	8.3 (7–9)	20.5 (20–21)	19.8 (19–21)	19.0 (15–24)	15.5 (15–16)	15.0 (13–16)	11.5 (9–14)
Average response rate (time point range)	85.9 (78.6–93.8)	91.0 (83.3–100.0)	80.3 (67.7–87.5)	94.0 (85.7–100.0)	70.6 (58.6–77.8)	96.9 (87.5–100.0)	84.6 (80.0–87.0)	89.0 (82.6–95.2)	89.9 (81.0–100.0)	79.6 (76.2–84.2)	88.6 (72.2–94.1)	86.6 (75.0–92.3)
**Qualitative interviews**												
Number invited	-^g^	6	-	8	-	-	10	10	-	-	-	-
Number participated	-	4	-	4	-	-	5	4	-	-	-	-

*LHD, Local health department; EBDM, Evidence-based decision making; EBPH, Evidence-based public health; EBI, Evidence-based interventions; FTE, Full-time equivalent; TA, Technical assistance; CHIP, Community health improvement plan*.

a *No ranges are provided at the group level to assure anonymity*.

b *Local health department full-time equivalent employee data from Missouri Department of Health and Social Services reported for calendar year 2017*.

c *Missouri Department of Health and Social Services 2018*.

d *Intervention phase commenced with the EBPH training and ended with the final quantitative survey collection*.

e *A 3.5 day in person EBPH course occurred when groups switched to intervention mode*.

f *Local health departments were supplied a menu of possible strategies to implement or could initiate other strategies. Reported are what was chosen and implemented. Those not chosen are not included*.

g *Not selected for qualitative interviews*.

### Quantitative Survey

**Figure 2:**
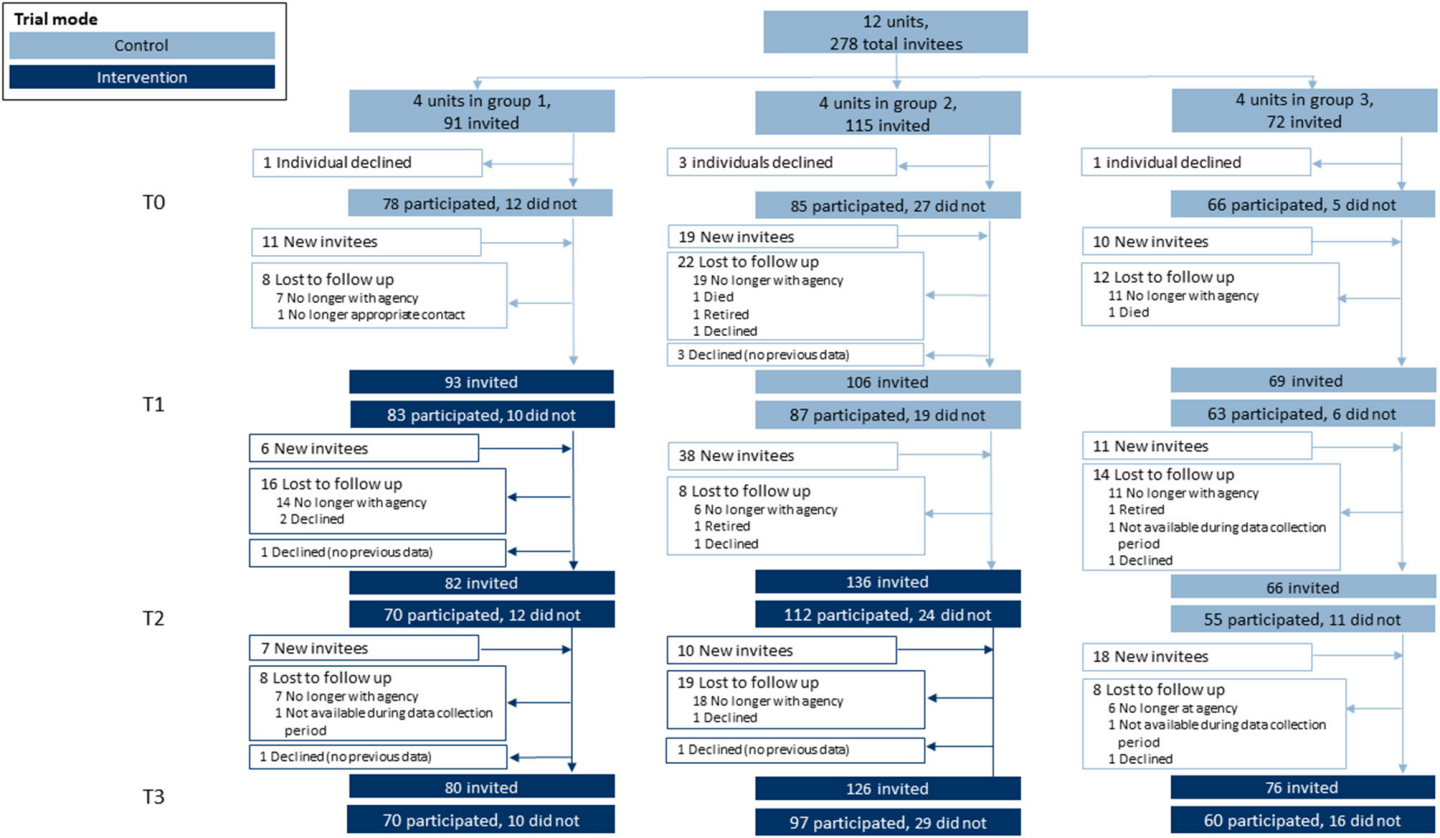
Participation flow diagram. This stepped-wedge design featured 12 units (local health departments) randomly assigned into one of three groups. Within each unit, individuals were invited to participate in a quantitative survey at four separate time points. Each time point included returning survey invitees and newly-invited individuals (open cohort design) where turnover warranted replacements with new hires.

Each LHD developed a list of key staff involved in chronic disease prevention who were invited to complete an online quantitative survey every 8 months during the entirety of the study (26 months). Participants were invited via email and reminded by both email and phone to boost response. Surveys were timed with group cross-over in order to collect control measures before each intervention period. We worked with LHDs to identify individuals to replace participants who were no longer at their respective organization (open cohort design) as shown in Figure 2.

Constructs assessed with the quantitative survey include three main areas of trial outcomes. First was competency for EBDM. Ten skills were assessed and align closely with objectives from the EBPH training that each LHD received (listed in **Tables 3, 4**). Importance and availability of each skill was assessed on an 11-point ordered scale. Participants selected from a list of eight evidence-based programs or policies related to chronic disease prevention which were currently implemented at their respective LHD. Twenty-two survey items assessed organizational culture supportive of EBDM processes on a 7-point Likert scale (1 = strongly disagree to 7 = strongly agree). The survey also assessed participant characteristics such as education level, length of employment in public health and at their current agency, gender identity, age, race and ethnicity. The full survey is available in Supplementary Material 2.

### Qualitative Interviews

The purpose of qualitative interviews was to understand how LHDs supported evidence-based processes after the initiation of the intervention phase of the study (i.e., how processes were implemented, any impacts, barriers and facilitators that influenced implementation, advice to other LHDs wanting to replicate). We selected four of the 12 LHDs based on trial randomization group, intervention adoption information, and raw change in outcome measures from baseline to final time point (Table 1). Selection in this manner was purposeful to represent LHDs with at least 16 months of intervention time prior to interview, active (high contact) participation in the intervention, and favorable outcomes from the raw quantitative data. The research team worked with each health promotion or chronic disease manager in the four LHDs to obtain leadership approval to participate in qualitative data collection. Participants were invited via email to complete audio-recorded phone interviews between October 2020 and January 2021.

The semi-structured interview guide, based on the team's previous work (24, 28), asked about organizational policies and procedures intended to support EBDM use, organizational environment and norms pertaining to EBDM, impact of instituted organizational policies and procedures on employees' day-to-day work, facilitators and challenges to integration of EBDM into day-to-day work, steps taken to sustain EBDM processes, recommendations for other LHDs, and recommendations for academic research teams for future studies with health departments. The full interview guide is provided in Supplementary Material 3.

### Analysis

Descriptive statistics for participant and LHD characteristics and main outcomes were compared across trial mode, or intervention and control groups. We calculated gaps in each skill for EBDM by subtracting availability from importance Likert rating (possible range of −10 to +10). An overall skill gap was created by taking the average across all 10 skill items. We summed all 8 possible EBIs to calculate an “EBI score” which had a possible range of 0–8. For organizational culture items, we grouped and averaged Likert items within six main focus areas based on previous work: awareness of culture supportive of EBDM, capacity and expectations for EBDM, resource availability, evaluation capacity, EBDM climate cultivation, and partnerships to support EBDM (20). A confirmatory factor analysis using data from all four time points according to standard procedures (29–31) demonstrated adequate fit and strict measurement invariance of the factor structure used for the national survey in Phase 1 (20). We used linear mixed-effect regression models for each outcome with LHD and Participant entered as random intercepts, trial mode (control or intervention) as fixed effect, and time as a categorical fixed effect. Public health degree, years worked in the public health field, job position category and accreditation status were entered as fixed effects. Kenward-Rogers approximations were used to determine significance of fixed effects, a common approach in fitting restricted maximum likelihood models in order to produce acceptable Type 1 error (32, 33). Where models violated assumptions of homoscedasticity, robust models were approximated. Survey data were managed and analyzed in R.

Each phone interview was audio-recorded, transcribed verbatim by rev.com, and de-identified. A deductive coding approach was used to analyze interview data. Two coauthors (RGP, SK) developed and refined a codebook. Co-authors (SK, RGP) independently coded 20% of the transcripts in NVIVO 12 qualitative software and then met to reach consensus (agreement above 95% and Kappa of at least 0.70) on discrepancies and finalize the coding before one co-author (SK) coded the remaining transcripts (34). The coding team (SK, RGP, PA) conducted content analyses through a dual independent process (34–36). For each topic, co-author pairs (PA, SK, RGP) independently reviewed coded texts and made notes to identify themes and summarize content. Pairs then met and reached consensus on final themes and subthemes.

## Results

Table 1 provides descriptive information about the three groups of LHDs including the EBPH training timing for each group, follow-up support, and management practices each LHD implemented to support EBDM. Overall, Group 3 had a higher average number of full time equivalent employees (144.1) and larger average jurisdiction populations (414,600 people) compared to groups 1 and 2. All but one LHD met in person for planning strategies. The first group averaged 8.5 follow-up meetings with the research team (over 24 months), the second group 9.5 over 16 months, and 2.5 for the third group over 8 months in the intervention phase.

### Quantitative Results

Overall, 519 LHD staff were eligible and invited to complete quantitative surveys during control periods and 593 during intervention (365 total unique individuals). A total of 434 completed during control and 492 during intervention resulting in 83.6 and 83.0% response, respectively. For each time period, the LHDs averaged 23.2 eligible staff (range 8.5–65.8) and 19.3 staff (range 8.3–46.8) which completed surveys. Of all participants (Table 2), most were female (82.1% control, 83.8% intervention), about half had at least a master's degree (51.7% control; 49.7% intervention), and less than a quarter completed a formal degree program in public health (20.3% control; 19.6% intervention). For job position, program managers or coordinators made up approximately one-third of participants (36.3% control; 34.2% intervention). Participants worked in public health for a little more than 10 years on average (mean = 10.7, SD = 9.7 control; 10.6, SD 9.3 intervention), about double the time they worked in their current organization (mean = 5.4, SD = 7.0 control; 5.64, SD 7.0 intervention).

**Table 2:** Sample characteristics at individual level by trial mode.

**Participant characteristic**	**Control** **(*N* = 433) N (%) or Mean (SD)**	**Intervention** **(*N* = 489)** **N (%) or Mean (SD)**
At least master's degree	224 (51.7%)	243 (49.7%)
Formal degree in public health (MPH, DrPH)	88 (20.3%)	96 (19.6%)
Years worked in public health field	10.67 (9.66)	10.64 (9.33)
Years worked at current organization	5.38 (7.00)	5.64 (7.00)
**Position**		
Executive/Director/Administrator	44 (10.2%)	43 (8.8%)
Program manager or coordinator	157 (36.3%)	167 (34.2%)
Technical expert	125 (28.9%)	135 (27.6%)
Other^a^	107 (24.7%)	144 (29.4%)
**Age**		
≤ 29 years	103 (23.8%)	92 (18.9%)
30–39 years	111 (25.7%)	127 (26.1%)
40–49 years	88 (20.4%)	100 (20.5%)
50–59 years	88 (20.4%)	116 (23.8%)
≥60 years	42 (9.7%)	52 (10.7%)
**Sex**		
Female	353 (82.1%)	403 (83.8%)
Male	77 (17.9%)	78 (16.2%)

a *Examples of other positions include health educator, nurse practitioner, and nutrition specialist*.

**Figure 3:**
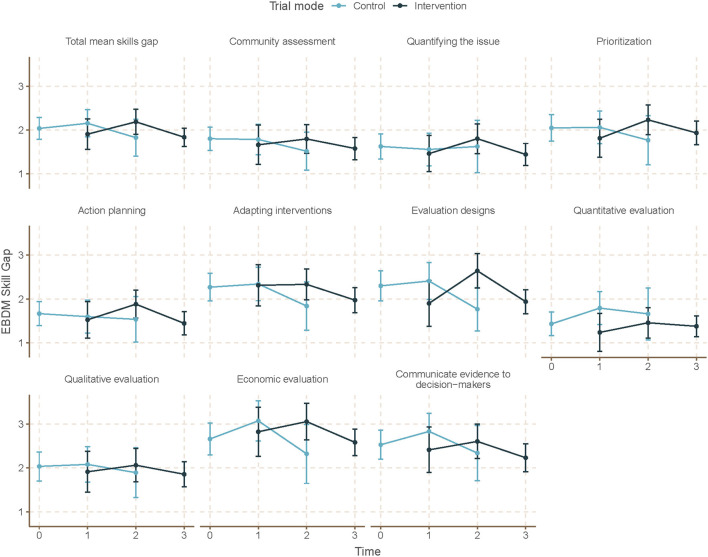
Mean EBDM skill gaps by time and trial mode. At each time point, mean and 95% confidence intervals for skill gaps in evidence-based decision making (EBDM) are displayed for individuals during control and intervention phases. EBDM skill gaps come from importance and availability of 10 skills measured on an 11-point ordered scale. For each skill, a skill gap was calculated by subtracting the availability rating from the importance rating. Time 0 represents baseline where all units (local health departments) were in control period. Time 3 is the final data collection point and all individuals are in intervention period. Total mean skill gap score was created as an average of the 10 individual competency gaps.

**Figure 4:**
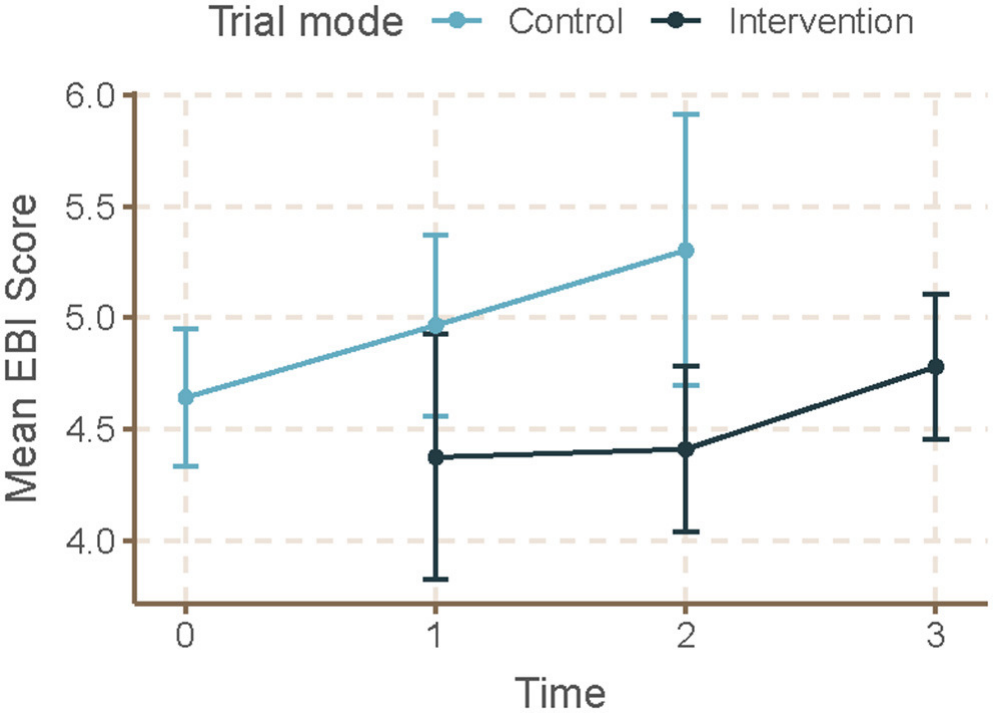
Mean EBI score by time and trial mode. At each time point, mean and 95% confidence intervals for evidence-based interventions (EBI) score are displayed for individuals during control and intervention phases. For EBI Score, participants selected from a list of eight evidence-based programs or policies related to chronic disease prevention which were currently implemented at their respective local health department. We summed all 8 possible EBIs to calculate the EBI score which had a possible range of 0–8. Time 0 represents baseline where all units (local health departments) were in control period. Time 3 is the final data collection point and all individuals are in intervention period. Total mean skill gap score was created as an average of the 10 individual competency gaps.

**Figure 5:**
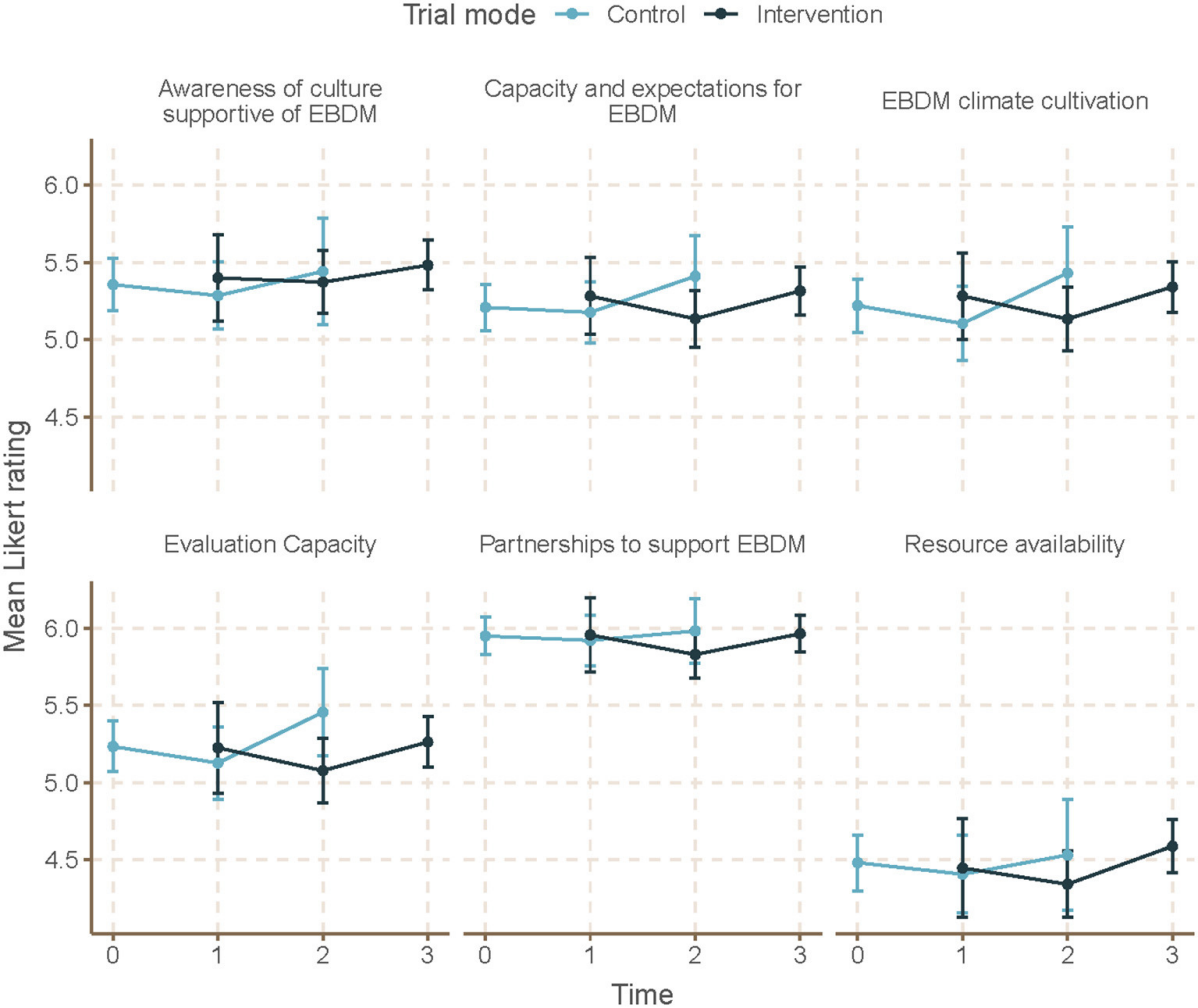
Mean EBDM culture items by time and trial mode. At each time point, mean and 95% confidence intervals for organizational culture supportive of evidence-based decision making (EBDM) items are displayed for individuals during control and intervention phases. All items on organizational culture supportive of EBDM were measured on a 7-point Likert scale. A summary score was created as an average of the items within each domain. Time 0 represents baseline where all units (local health departments) were in control period. Time 3 is the final data collection point and all individuals are in intervention period. Total mean skills gap score was created as an average of the 10 individual competency gaps.

Mean skill gaps in EBDM (mean of all 10 skill gaps) were similar for both control and intervention trial modes (2.05 95% CI 1.87–2.23 control; 1.98 95% CI 1.82–2.13 intervention) (Table 3). Mean skill gaps in EBDM (and 95% CIs) for both intervention and control groups at each time point are displayed in Figure 3. Economic evaluation was the largest skill gap (2.76 95% CI 2.50–3.02 control; 2.80 95% CI 2.57–3.02 intervention) followed by communicating evidence to decision-makers (2.61 95% CI 2.37–2.85 control, 2.40 95% CI 2.17–2.62 intervention). More EBIs were reported during the control period (4.84 95% CI 4.61–5.07 control, 4.58 95% CI 4.36–4.80 intervention). Mean EBI scores (and 95% CIs) for intervention and control groups at each time point are displayed in Figure 4. For both control and intervention, partnerships that support EBDM had the highest mean Likert scale rating among other organizational culture items (5.94 95% CI 5.86–6.03 and 5.91 95% CI 5.83–6.00, respectively). The lowest mean Likert ratings for both were in resources available to support EBDM (4.46 control and 4.47 intervention, respectively). Mean organizational culture for EBDM items (and 95% CIs) for intervention and control groups at each time point are displayed in Figure 5.

**Table 3:** Outcome measures by trial mode.

	**Control (*N* = 433) Mean (95% CI)**	**Intervention** **(*N* = 489)** Mean **(95% CI)**
**Competency gaps in EBDM** ^a^		
Mean sum gap score^b^	2.05 (1.87, 2.23)	1.98 (1.82, 2.13)
Gap-community assessment	1.76 (1.57, 1.95)	1.67 (1.49, 1.86)
Gap-quantifying the issue	1.60 (1.39, 1.81)	1.58 (1.39, 1.76)
Gap-prioritization	2.02 (1.80, 2.23)	2.02 (1.83, 2.21)
Gap-action planning	1.62 (1.42, 1.83)	1.62 (1.43, 1.80)
Gap-adapting interventions	2.24 (2.02, 2.46)	2.16 (1.96, 2.36)
Gap-evaluation designs	2.27 (2.03, 2.51)	2.19 (1.98, 2.40)
Gap-quantitative evaluation	1.59 (1.38, 1.79)	1.38 (1.20, 1.57)
Gap-qualitative evaluation	2.03 (1.80, 2.27)	1.94 (1.73, 2.15)
Gap-economic evaluation	2.76 (2.50, 3.02)	2.80 (2.57, 3.02)
Gap-communicating evidence to decision-makers	2.61 (2.37, 2.85)	2.40 (2.17, 2.62)
**Implementing evidence-based programs** ^c^		
EBI score (min: 0, max: 8)	4.84 (4.61, 5.07)	4.58 (4.36, 4.80)
**Organizational culture that supports EBDM (Raw)** ^d^		
Awareness of culture supportive of EBDM (# items)	5.34 (5.22, 5.47)	5.43 (5.31, 5.54)
Capacity and expectations for EBDM (# items)	5.22 (5.11, 5.33)	5.24 (5.14, 5.35)
Resource availability (# items)	4.46 (4.32, 4.60)	4.47 (4.35, 4.60)
Evaluation capacity (# items)	5.23 (5.10, 5.35)	5.19 (5.07, 5.31)
EBDM climate cultivation (# items)	5.21 (5.08, 5.34)	5.26 (5.14, 5.37)
Partnerships to support EBDM (# items)	5.94 (5.86, 6.03)	5.91 (5.83, 6.00)
**Organizational culture that supports EBDM (Factor)** ^e^		
Awareness of culture supportive of EBDM (# items)	−0.02 (−0.11, 0.07)	0.01 (−0.07, 0.10)
Capacity and expectations for EBDM (# items)	−0.01 (−0.10, 0.08)	0.01 (−0.08, 0.09)
Resource availability (# items)	−0.00 (−0.09, 0.09)	0.00 (−0.08, 0.08)
Evaluation capacity (# items)	0.01 (−0.08, 0.10)	−0.01 (−0.09, 0.08)
EBDM climate cultivation (# items)	−0.00 (−0.09, 0.09)	0.00 (−0.08, 0.09)
Partnerships to support EBDM (# items)	0.02 (−0.07, 0.10)	−0.02 (−0.10, 0.07)

*EBDM, Evidence-based decision making; EBI, Evidence-based interventions*.

a *Competency Gaps come from importance and availability of 13 skills measured on an 11-point ordered scale. For each skill, a skill gap was calculated by subtracting the availability rating from the importance rating*.

b *A mean skill-gap score was created as an average of the 13 individual competency gaps*.

c *EBI Score- Participants selected from a list of eight evidence-based programs or policies related to chronic disease prevention which were currently implemented at their respective LHD. We summed all possible EBIs to calculate an “EBI score” which had a possible range of 0–8*.

d *All items on organizational culture supportive of EBDM were measured on a 7-point Likert scale. A summary score was created as an average of the items within each domain*.

e *Factor scores for organizational culture support domains were derived from confirmatory factor analysis*.

After accounting for clustering by LHD and repeated response in mixed-effects linear models, we found no significant time or intervention effect with regard to EBDM skill gaps (Table 4). No net intervention effect was found in implementing EBIs, though the last time point had significantly more EBIs implemented overall compared to the first time point (0.57, 95% CI 0.01–1.14, *p* < 0.05). Two organizational culture support for EBDM factor scores were significantly reduced for intervention: EBDM awareness (−0.14, 95% CI −0.26 to −0.01, *p* < 0.05) and climate cultivation (−0.14, 95% CI −0.27 to −0.02, *p* < 0.05). At the final collection (time 4), significantly larger factor scores were found in awareness (0.20, 95% CI 0.04–0.35, *p* < 0.05), EBDM capacity and expectations (0.21, 95% CI 0.04–0.35), evaluation capacity (0.17, 95% CI 0.01–0.33), and climate cultivation (0.19, 95% CI 0.01–0.37) compared to the first time point.

**Table 4:** Intervention effect estimates for outcomes.

	**Intervention Estimate (95% CI)**	**Time 1 Estimate** **(95% CI)**	**Time 2 Estimate (95% CI)**	**Time 3 Estimate** **(95% CI)**
**Competency gaps in EBDM** ^a^				
Mean sum gap score^b^	0.00 (−0.26 to 0.27)	−0.01 (−0.22 to 0.23)	0.00 (−0.29 to 0.29)	−0.19 (−0.54 to 0.15)
Gap-community assessment	−0.05 (−0.47 to 0.36)	−0.04 (−0.39 to 0.30)	−0.13 (−0.57 to 0.32)	−0.19 (−0.71 to 0.34)
Gap-quantifying the issue	−0.08 (−0.52 to 0.36)	−0.05 (−0.41 to 0.32)	0.16 (−0.32 to 0.63)	−0.08 (−0.64 to 0.48)
Gap-prioritization	0.06 (−0.36 to 0.47)	−0.08 (−0.42 to 0.27)	−0.05 (−0.51 to 0.40)	−0.21 (−0.74 to 0.32)
Gap-action planning	0.20 (−0.20 to 0.60)	−0.15 (−0.48 to 0.18)	−0.06 (−0.49 to 0.37)	−0.41 (−0.92 to 0.09)
Gap-adapting interventions	0.13 (−0.30 to 0.55)	−0.01 (−0.37 to 0.34)	−0.17 (−0.63 to 0.29)	−0.39 (−0.93 to 0.15)
Gap-evaluation designs	−0.03 (−0.52 to 0.45)	−0.10 (−0.50 to 0.30)	0.09 (−0.44 to 0.61)	−0.34 (−0.95 to 0.28)
Gap-quantitative evaluation	−0.28 (−0.72 to 0.15)	0.22 (−0.14 to 0.58)	0.20 (−0.27 to 0.66)	0.20 (−0.34 to 0.75)
Gap-qualitative evaluation	0.13 (−0.33 to 0.59)	−0.12 (−0.50 to 0.26)	−0.20 (−0.70 to 0.29)	−0.34 (−0.92 to 0.24)
Gap-economic evaluation	0.30 (−0.17 to 0.78)	0.18 (−0.22 to 0.57)	0.01 (−0.51 to 0.52)	−0.34 (−0.94 to 0.26)
Gap-communicating evidence to decision-makers	0.13 (−0.35 to 0.61)	0.08 (−0.31 to 0.48)	−0.17 (−0.68 to 0.35)	−0.50 (−1.11 to 0.11)
**Implementing evidence-based programs** ^c^				
EBI score (min: 0, max: 8)	−0.23 (−0.67 to 0.22)	0.32 (−0.05 to 0.69)	0.47 (−0.02 to 0.95)	**0.57 (0.01 to 1.14)**
**Organizational culture that supports EBDM** ^d, e^				
Awareness of culture supportive of EBDM (3 items)	**−0.14 (−0.26 to** **−0.01)**	−0.01 (−0.11 to 0.09)	0.11 (−0.02 to 0.25)	**0.20 (0.04 to 0.35)**
Capacity and expectations for EBDM (7 items)	−0.12 (−0.25 to 0.01)	0.01 (−0.10 to 0.12)	0.11 (−0.03 to 0.25)	**0.21 (0.04 to 0.37)**
Resource availability 3 items)	−0.07 (−0.20 to 0.07)	−0.02 (−0.13 to 0.09)	0.00 (−0.14 to 0.15)	0.14 (−0.03 to 0.32)
Evaluation capacity (3 items)	−0.11 (−0.25 to 0.02)	−0.01 (−0.12 to 0.10)	0.10 (−0.04 to 0.25)	**0.17 (0.01 to 0.33)**
EBDM climate cultivation (3 items)	**−0.14 (−0.27 to** **−0.02)**	−0.03 (−0.14 to 0.07)	0.09 (−0.05 to 0.22)	**0.19 (0.01 to 0.37)**
Partnerships to support EBDM (3 items)	−0.02 (−0.16 to 0.12)	−0.05 (−0.16 to 0.07)	−0.05 (−0.20 to 0.10)	0.02 (−0.16 to 0.20)

*Bold for p < 0.05 based on Kenward-Rogers (Kr) approximations, or where presence of heteroskedasticity was detected, robust estimation. Models include PH degree, years in PH, job position, FTEs, and accreditation as fixed effects. Time 0 (or baseline where all 12 units were in control period) was treated as reference category for time*.

*EBDM, Evidence-based decision making; EBI, Evidence-based interventions*.

a *Competency Gaps come from importance and availability of 10 skills measured on an 11-point ordered scale. For each skill, a skill gap was calculated by subtracting the availability rating from the importance rating*.

b *A mean skill-gap score was created as an average of the 10 individual competency gaps*.

c *EBI Score- Participants selected from a list of eight evidence-based programs or policies related to chronic disease prevention which were currently implemented at their respective LHD. We summed all 8 possible EBIs to calculate an “EBI score” which had a possible range of 0–8*.

d *All items on organizational culture supportive of EBDM were measured on a 7-point Likert scale. A summary score was created as an average of the items within each domain*.

e *Factor scores for organizational culture support domains were derived from confirmatory factor analysis*.

### Qualitative Interviews

A total of 17 interviews were conducted with LHD staff in the trial, representing 4 of the 12 LHDs. Of those interviewed, most were white (71.0%), mostly non-Hispanic (94.0%) and worked an average of 6.7 years at their current agency. Several themes emerged from interviews with LHD staff participants.

#### Facilitators

Management practices, or the way in which various mechanisms within LHDs operate to either challenge or support EBDM including leadership, organization culture, workforce development, agency/system planning, program reviews, partnerships, and accreditation processes/status were facilitators of EBDM processes within LHDs (Table 5). Leadership activities that were most helpful to support EBDM included dedicating staff, creating specific guidelines, setting expectations for use, and providing trainings, resources and guidance. A culture shift, including dialogue, helped to change the “atmosphere” and “ingrain” EBDM implementation into LHD processes. Specific workforce development practices such as staff-led training sessions helped to “*introduce new staff to what that [EBDM] is and our processes and what we do.”* Incorporating EBDM into job descriptions and performance reviews to introduced a level of accountability to “*continually working on that [EBDM].”* Agency level plans included strategic planning that included EBDM and including EBDM into performance management systems and community health improvement plan development. Also helpful to support EBDM within LHDs was the incorporation of programmatic reviews to align programs with LHD priorities, find evidence-based approaches, compare programs with EBDM process, and enhance or initiate program evaluation. In working with partners, LHDs provided staff time to support partner EBDM use and also facilitated use of EBDM through the CHIP process. Management practices put in place to apply for or maintain PHAB accreditation also helped to support EBDM implementation and served as a “*driver*” to continue EBDM practices.

**Table 5:** Barriers and facilitators to use of evidence-based decision making in local health departments.

**Domain**	**Description**	**Illustrative quote**
**Barriers**		
Staff turnover	Constant stream of new employees to replace those that leave the LHD	“The main challenges were just confusion across the board. It was a new system, still is somewhat new, because obviously we have turnover, and so with new employees, you have to explain just like it was the first time they've ever seen it because it is.”
Limited EBDM training	Few staff directly trained in EBDM	“If you'd bring that in-person training to the local health departments individually, and maybe to train as many staff as possible, in a one-stop-shop, so everybody hears the same thing.” “A lot of the information was passed through several layers, and by the time it got to staff they weren't quite sure what to do with it or what the expectation was, or how it would be helpful to them.”
	EBDM overwhelming to those without public health background	“So we have involved staff, management level staff in the process. For about half the staff, this was new to them. So this was overwhelming. So that was a challenge. So, I think just education with them and I've worked a lot with them one-on-one to increase knowledge and showing how these things can be used.” “I think I had some staff who came through it [EBDM training] and I was surprised that they didn't learn more skills, because the content was there, but I think it was just that they were learning so much and they didn't have the opportunity to put it into practice before they learned something new.” [recommended ongoing smaller trainings]
Limited staff engagement	Engagement in EBDM waned over time in some LHDs	“We attended a training in St. Louis, and right after that occurred, we were really involved in follow-up conversations and ideas for how we were going to take what we learned at the training and actually implement it and what that was going to look like. And then I feel like that just went away…I was individually able to take what I had learned and apply it, but as a whole, as the health department I don't think it was broadly implemented…It just kind of dropped off.” “That stalling that would happen so much or occasionally.”
Competing or changed priorities	Competing priorities	“Number one challenge right now is COVID. So that has slowed down progress substantially from where we wanted to be.”
	Changing priorities	“Really just the shifting of priorities, I guess, is really the only kind of challenges. But those will always be there.”
Cost	Lack of budget for evidence-based interventions in chronic disease prevention	“I think another challenge is we would sometimes find something too and that's great but the cost of that is something that we don't have the money to afford or to even start to address. When it was a cost issue, that just kind of stopped things. There really was no budget to do most things, and so whenever that challenge arose, I think we were just kind of stopped.”
Staff pushback	Initial hesitancy or reluctance to use EBDM	“We had some employees early on that pushed back. They were looking at it as just another fad type of thing or they thought maybe it was a way to get rid of programs and things like that. And what we did was re-educate and that pretty much worked. I mean, we just kept readdressing it.”
Reactions to change	Resistance to change	“The other challenges are just staff who are resistant to change or resistant to new ideas. As far as managing that, it is essentially we are incorporating things like making staff accountable for integrating equity into performance evaluations.”
	Too much change creates confusion	“The foundation remains the same, but if we find a better way to do something we're not hesitant to make a change. So that's another thing that's a challenge at times. When you change things too much, then it just becomes confusing for everyone.”
**Facilitators**		
Distributed leadership	Leaders expected staff to use EBDM	“Leadership and their department heads set the bar high for themselves. So they're not going to expect anything less from us.” [Required protocol review]: “It makes people think before they adopt a program. There's more thought behind it. More research that goes into it and evaluation planning that goes into it prior to adoption as well…I think adapting the protocol so it's part of our department requirements is probably going to be the one that is the most long-lasting.”
	Managers provided additional trainings and guidance	“So my superiors sat me down to make sure I understood what evidence-based programming was and examples of evidence-based programs, and if there wasn't evidence readily available, how to get it. So, thus the learning to fly at the same time I'm flying. Learning to fly and building the plane…Our health department folks will jump through hoops to make sure we have what we need to stay excellent and stay evidence-based.”
	Leaders dedicated staff for EBDM	“Having a specific team that is involved in that, and that's what we do. I think that has helped because it takes the burden off those supervisors to do that.”
	Created guidelines for EBDM rollout	“I think the most useful was just several of us getting into a room and just kind of talking about how we're going to roll this out.” “I think that foundational conversation is what led to the ability to get there and for people to get onboard.”
EBDM knowledge		“So that was good, knowing that we have quite a few staff that are receptive to the process, they know what evidence based decision making is, they know how to use it.”
Incremental changes		“I think no matter how slow the process begins, you just have to integrate on whatever level you have. Start small and keep moving forward.” “Starting in one program or one division and really putting in the energy…being able to find the cheerleaders or the people that are willing to take on that work and to help lead it and then also to continually look back at it and how do we expand.”
Aligned vision and action		“Everything that we do, we make sure it relates back to our strategic plan and our strategic plan is based on EBDM.” “Public health reaccreditation is…really the driver behind us maintaining that [EBDM], that foundation and still having the intent of driving it forward after of course we are done responding to a pandemic.” “Having a good foundation in the beginning, making sure that everybody's on the same page as far as your mission and goals.”
Collaborative relationships		“Having people that have the same understanding or are in the same frame of mind when it comes to EBDM and it's part of what they do, that made things easier for us.”
Culture shift	EBDM ingrained into LHD's organizational culture/climate	“Looking back from how far we've come, I would say that we have definitely seen a culture shift, and seen more and more of those conversations around evidence-based decision making…It's been very successful and we definitely have seen a change in the overall atmosphere of the health department since the start of that culture shift.” “It's [EBDM] ingrained in our culture.”
Information access	Access to data	“So when you can view the data easily, it becomes part of meetings and you start to make those decisions based on what the data is telling you.”
	Access to step-by-step guidelines	[One LHD created an EBDM manual early on.] “Having the tool itself made it easier, but it also helped generate support essentially for the initiatives.”
	Access to programmatic examples	“We've just taken the time to do more research on what evidence-based practices other health departments are doing.”

*EBDM, Evidence-based decision making; LHD, local health department*.

#### Barriers

Despite management practices that were described as facilitators, several challenges to EBDM processes were also discussed by LHD staff (Table 5). Staff turnover, small numbers trained, time to fully implement changes, not enough support, and low level of involvement after training all were mentioned as barriers to moving EBDM management practices further. Staff turnover was described as especially disruptive to continuing with EBDM work, “*we'll get going for a little while... we'll have some turnover or something will happen where we have to shift our focus and then it can be difficult sometimes sort of going back and saying, okay, how can we get, how can we keep that momentum going?”* Staff turnover also makes it hard to keep enough staff trained in EBDM, “*our health department had a whole bunch of people that went through the EBDM training, but most of them have left now…But if there was an opportunity to continue offering the EBDM trainings, just because of that staff turnover, that would be one resource that would be amazing.”* Time to develop and implement EBDM practices was challenging as one participant described because “*in public health agencies, there's never going to be enough time.”* The participant stated that LHDs should “*start small and just keep moving forward.”* Another participant described how implementing EBDM processes after the training “*took a backseat because it did take time and effort and staffing in order to develop a plan, continue training, implement it, evaluate, et cetera.”* In terms of needing additional support, participants wanted more concrete examples and continued technical assistance that was more formalized. According to one participant, when the research team did “*…provide us with really concrete steps or examples, that was really helpful. So for example, some of the website structure that actually had the list of evidence-based programs that we could then kind of replicate here. Or walking us through how to do a logic model. The evaluation series that they offered us, the online trainings that were made available, those were super helpful. So really just kind of that, that concrete examples and technical assistance is helpful.”*

## Discussion

Overall, our mixed methods study highlights the challenge of complex interventions and their implementation with heterogeneous organizations. While the quantitative outcomes were not significantly improved in our analysis, the qualitative data highlight challenges and future directions for continued efforts in building EBDM capacity.

We found no intervention effect on skills for EBDM. In a similar randomized control trial with 12 state health departments, Brownson et al. (9) found significant reductions in EBDM skill gaps in 6 skill areas (adapting interventions, prioritization, quantifying the issue, communicating to policy makers, community assessment, and qualitative evaluation) at 18–24 months post-training. These improvements were found among the primary intervention group (or those who had attended the state-based EBPH training). With smaller local health departments and training sizes, it was not possible to complete similar analyses, though it is possible we may have seen improvements among the smaller group of those who attended the EBPH training. Perhaps direct training linked with individual skill-building may not diffuse to others in the department unable to attend or that are brought on later during an extended study time. As such, continuous training opportunities, possibly embedded into health department work flows, are needed. In our study, interview participants wanted additional direct training in how to apply EBDM principles in their day-to-day work, such as examples of evidence-based interventions other LHDs were using and additional hands-on support with program evaluation.

Trial mode was associated with significant reductions in organizational culture factor items including awareness, evaluation capacity, and climate cultivation for EBDM. Brownson et al. (9) found that, except for access to evidence and skilled staff, the remaining three organizational culture factors were not changed for the primary intervention group with negative, nonsignificant estimates in supervisory expectations and in participatory decision-making. In both studies, organizational readiness (and/or the readiness to engage in participatory research) was not comprehensively assessed. It is possible that while we looked for changes in organizational culture supportive of EBDM, perhaps we missed important contextual influences affecting implementation of the intervention (37–39). In a review, Willis et al. (39) outlined six guiding principles that influence the sustainability of organizational change: aligned vision and action; incremental changes within a comprehensive transformation strategy; distributed leadership; staff engagement; collaborative relationships; and continuous assessment and evaluation of change. Many of these principles were mentioned qualitatively by LHD staff as strategies that were helpful in shifting processes to be more EBDM-focused. Determining additional ways to measure incremental changes and/or measures that capture the changes that happened from starting “small and just keep moving forward” could help explain how change happens and is sustained in LHDs.

Implementing and sustaining organizational change through staff engagement is further complicated when faced with the instability of the public health workforce. Maintaining a stable workforce is a well-documented challenge in public health (40, 41) and is compounded by local health departments with smaller budgets and fewer employees (7, 42). Similar challenges were mentioned in a different mixed method study with 31 LHDs in New York (43). Sosnowy et al. (43) found that EBDM philosophy was generally understood and supported within health departments, but operationalizing its concepts was challenged by limited funding, staff and resources. Turnover was commonly mentioned by interviewees and also confirmed by the study team at each data collection point in terms of updating tracking sheets and contact information to account for staffing changes. Every time a trained staff member leaves, a new staff must be on boarded and trained. This is disruptive to keeping “*the momentum going*” on work to change management policy and/or practices. However, strong and determined leadership may be resilient in the face of turnover and limited resources, as was found in our study and in several other studies exploring supports for evidence-based processes (24, 43, 44). In addition, embedding standards for the use of EBDM into daily departmental activities creates structure, a level of capacity, and efficiency needed where high turnover is present.

Interviewees described how the focus on changing policy and management practices that occurred directly after training began to wane over time. Expansion and lag periods make capturing change difficult and reinforces the need for complex interventions with public health departments to account for such context (45, 46). LHDs were the main drivers of the intervention, from the staff who attended the trainings, to the approaches to focus on, and the level of engagement with the research team. Engagement varied across the LHDs (Table 1) and even LHDs working closely with our research team cited various barriers to moving EBDM processes along via interviews. This reinforces the need for participatory research with practitioners to depart from the notion that the research pipeline is evidence “delivered” to practitioners (47). A promising approach involves Academic Health Departments (AHDs), or formal or informal arrangements between governmental public health agencies and academic institutions with the overall goal of shared benefits through research, practice, and development of the next generation of the public health workforce (18, 48). AHDs offer sustained partnerships as a main benefit and provide infrastructure to manage organizational culture shifts, which demand long-term commitment. AHDs have been shown to implement more EBDM processes as compared to non-AHDs (18), underscoring the still forming AHD research agenda, which maps to various concepts of EBDM (49, 50). Learning “*what works*” from other LHDs in forming supportive and efficient AHD partnerships benefits the overall movement toward increasing such partnerships, but external funding for such coordination is limited (51). Our study and the accompanying body of work can be used to inform efforts.

## Limitations

The current study is limited to LHDs in Missouri. LHDs are governed differently in each state and even locally, which has unique implications for building EBDM capacity. Responses to the quantitative survey were self-reported, which introduces the possibility of response bias. Evidence-based programs and policies reported by each individual for their LHD were not verified beyond self-report. Similarly, interview participants were informed of their anonymity, but self-censure may still be a potential limitation. Our final wave of data collection began in February 2020, when our LHDs were in the beginning stages of responding to the COVID−19 pandemic, which challenged data collection. We saw a drop in response rates for the final wave of data collection period, especially with the third group/wave (those who received intervention in the last period). The pandemic response was another reason we did not conduct interviews with more LHDs. Our purposive sampling approach allowed the research team to obtain rich contextual information from LHDs with mid- to high levels of participation while also minimizing disrupting or burdening all 12 LHDs during a demanding period addressing the COVID−19 pandemic in their communities. It is possible that key participants were uninvolved in the last part of our intervention period. Finally, the stepped-wedge design has several practical limitations that are trade-offs for robustness such as data collection intensity and burden, but allows for all 12 LHDs to be in the intervention group. For example, each participant received four surveys within the 26 month trial period. Staff may have been unable to respond, and therefore, we potentially missed their perspective.

## Conclusions

LHDs are unique in that they represent the frontline of chronic disease prevention, localized with the ability to engage the communities they serve in evidence-based programs and policies. Our study and related literature serve in understanding how best to build capacity within LHDs to support EBDM processes. LHDs can facilitate integration of EBDM into day-to-day public health practice through leadership support, by fostering a supportive organizational climate and culture, and by embedding EBDM steps into internal written plans, policies, and standardized procedures. Future directions should focus on sustained partnerships that keep LHDs in the driver's seat and give consideration for known challenges (e.g., turnover, limited funding).

## Data Availability Statement

The datasets analyzed during the current study are available from the corresponding author upon reasonable request.

## Ethics Statement

The studies involving human participants were reviewed and approved by Washington University in St. Louis Institutional Review Board (#201705026 and #202010031). The Ethics Committee waived the requirement of written informed consent for participation.

## Author Contributions

RP led intervention planning and implementation, quantitative and qualitative data collection, and data management. PA assisted with intervention planning and implementation, quantitative data collection, and qualitative data analysis. RB led the study conceptualization and design and assisted with intervention planning and implementation. YY assisted with study design, data analysis, and statistical support. RJ assisted with data management and led data analysis and manuscript development. SK supported both quantitative and qualitative data collection and coding and analysis. DD assisted with survey tool development, recruitment, and interpretation of results. SM supported quantitative data analyses. All authors contributed to manuscript development and approved the final version.

## Funding

This study was funded by the National Institute of Diabetes and Digestive and Kidney Diseases of the National Institutes of Health (Award Nos. R01DK109913, P30DK092949, and P30DK092950), the National Cancer Institute (Award No. P50CA244431), the Centers for Disease Control and Prevention (Award No. U48DP006395), and the Foundation for Barnes-Jewish Hospital.

## Author Disclaimer

The findings and conclusions in this article are those of the authors and do not necessarily represent the official positions of the National Institutes of Health or the Centers for Disease Control and Prevention.

## Conflict of Interest

SK was employed by RAND Corporation. DD was employed by National Association of County and City Health Officials. The remaining authors declare that the research was conducted in the absence of any commercial or financial relationships that could be construed as a potential conflict of interest.

## Publisher's Note

All claims expressed in this article are solely those of the authors and do not necessarily represent those of their affiliated organizations, or those of the publisher, the editors and the reviewers. Any product that may be evaluated in this article, or claim that may be made by its manufacturer, is not guaranteed or endorsed by the publisher.

## Supplementary Material

Supplementary Material 1 Menu of organizational supports for evidence-based decision making. This document was presented to units (local health departments) after crossing over to intervention period, following participation in the multi-day evidence-based public health training. The menu offers examples of strategies to support evidence-based processes within health departments.

Supplementary Material 2 Quantitative survey. The quantitative survey was administered at four separate time points over the course of the study. All individuals received the same survey tool regardless of the intervention phase of their respective unit (local health department).

Supplementary Material 3 Qualitative interview guide. These questions and prompts were used in interviews with local health department staff to guide a discussion around facilitators and barriers to implementing evidence-based processes within their respective agencies.
