# Supplementary material for: How to “Start Small and Just Keep Moving Forward”: Mixed Methods Results From a Stepped-Wedge Trial to Support Evidence-Based Processes in Local Health Departments
Source: Front Public Health. 2022 Apr 28;10:853791. doi: 10.3389/fpubh.2022.853791 (PMC9096224; doi:10.3389/fpubh.2022.853791)
Supplement: Supplementary Material 1 — Menu of organizational supports for evidence-based decision making. This document was presented to units (local health departments) after crossing over to intervention period, following participation in the multi-day evidence-based public health training. The menu offers examples of strategies to support evidence-based processes within health departments. [file Table_1.DOCX]

**
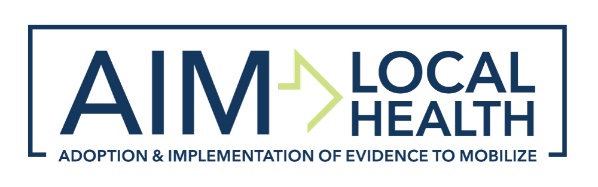

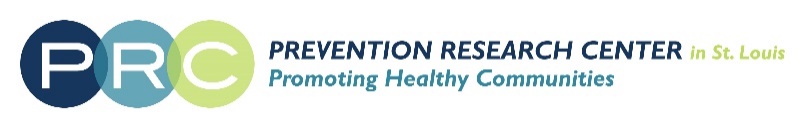
**

| **Menu of organizational supports for EBDM LHDs could select after cross-over to intervention and completion of EBPH training** | | |
| --- | --- | --- |
| **Area** | **Activity** | **Description** |
|  |  |  |
|  |  |  |
| Accreditation | Accreditation preparations | Local health assessment and plan, formalized decision making, documentation of evidence, documentation reviews, site visit, approval |
| Access to scientific information | Electronic targeted messages | Participants receive an email indicating a systematic review related to diabetes or chronic disease control is available – short summary of the research and actions that might be taken based on the evidence |
| Workforce development | Evidence-based public health (EBPH) training | In-person multi-day training in EBPH and evidence-based decision-making skills (in St. Louis), 9 modules, initial intervention (kick-off activity for intervention period) |
|  | Supplemental brief skill trainings | Provided by study team or consultant, in-person or webinar (e.g., evaluation skills) |
|  | Non-study national trainings | Hosted in-person EBPH and evidence-based decision-making skill trainings by national organizations and/or encouraged out-of-state training beyond those required by funders |
|  | Quality improvement | Quality improvement or performance management trainings, guidance |
|  | New employee orientation in evidence-based practice | Via archived webinars or course materials, facilitated discussions, meetings |
| Leadership, management supports | Chronic disease leadership teams expect evidence-based practice | Leaders and supervisors continually ask ‘what is the evidence’, communicate expectations to staff, champion evidence-based practice, encourage use of data for decision making, encourage skill building |
|  | Use of data for decision making | Use data to prioritize programs, develop work plans, and monitor progress; share performance measures, data on intranet or centralized data systems |
|  | Centralized data systems | Dashboard development to prioritize, measure, and track objectives and link to evidence base; share performance measures and data |
|  | Meetings incorporate | Work group and cross-section meetings address evidence-based practice, present evidence, plans; (in leadership and in training) |
|  | Performance reviews and EBDM | Work unit employee evaluations include objectives on evidence-based practice and decision-making learning and application |
|  | Hiring practices address EBDM | Job descriptions, interview questions address EBDM; hire people with public health competencies; hire specialty staff including evaluators and epidemiologists |
|  | Participatory decision making | Staff and partner input obtained, sharing of information for decision making |
|  | Common language for evidence-based practice | Creating and using common evidence-based practice and decision-making language across program areas |
|  | Administrative reorganization for coordination | Organizational restructuring at the group or division/section levels to increase coordination across programs and conduct joint projects across programs |
| Organizational changes | Evidence-based practice engrained | Evidence-based practice and decision-making an embedded inseparable aspect of day-to-day work; strong expectation from leadership; high priority |
|  | Learning orientation | Culture supports professional development and ongoing learning, providing links to webinars, bringing in guest speakers |
| Relationships and partnerships | Partnerships with in-state universities | Ongoing partnering for evaluation, trainings, internship placement |
|  | Partner technical assistance and training | Phone and in-person guidance for partners’ evidence-based work plans, evaluation, logic models; Provide evidence-based practice and decision-making trainings to partners |
|  | Relationship building | Active steps to build or maintain positive partner relationships with open communication, trust, mutual respect, ensuring partner engagement and coalition development |
| Financial practices | Performance-based contracting | Funded partners required to implement evidence-based approaches as prescribed or selected from a menu, with performance objectives, work plans, and evaluation; holding partners accountable for evidence-based interventions |
|  | Proposals approved internally for evidence-based practice prior to submission to funder | Local health department pre-approval process for grant applications to funders with requirements to show objectives, evidence basis, performance measures, evaluation plan |
