# Supplementary material for: How to “Start Small and Just Keep Moving Forward”: Mixed Methods Results From a Stepped-Wedge Trial to Support Evidence-Based Processes in Local Health Departments
Source: Front Public Health. 2022 Apr 28;10:853791. doi: 10.3389/fpubh.2022.853791 (PMC9096224; doi:10.3389/fpubh.2022.853791)
Supplement: Supplementary Material 2 — Quantitative survey. The quantitative survey was administered at four separate time points over the course of the study. All individuals received the same survey tool regardless of the intervention phase of their respective unit (local health department). [file Data_Sheet_1.pdf]

Adoption and Implementation of evidence to Mobilize Local Health (AIM-Local Health)
Quantitative Survey

Purpose: Ross Brownson and colleagues at the Prevention Research Center at Washington University in St. Louis are working on a project exploring strategies to enhance the use of evidence-based diabetes and chronic disease prevention and control among local health departments. The project name is AIM-Local Health (Adoption & Implementation of evidence to Mobilize Local Health), and is funded by the National Institutes of Health.

We are asking public health employees and staff at partnering agencies/organizations to help us by completing this survey. The findings will help us understand and assess strategies to support evidence-based efforts.

This survey will take approximately 15-20 minutes to complete. Your responses are anonymous and participation is voluntary. Survey responses are not linked to anyone's name or email address. Only summary findings will be reported that do not identify people or organizations.

To thank you, we are offering everyone who completes the survey an optional $20 Amazon gift card. The gift card is not required to participate in the survey. At the end of the survey, you will be redirected to an optional separate webpage where you can choose to accept the gift card.

For additional information about the study purpose, procedures, risk and protections, please go to: [link to IRB study information sheet].

I understand my participation in this survey is voluntary, and my answers are anonymous.
- ❍ Yes, I would like to take this survey
- ❍ No, I prefer not to take this survey [If selected, skip to the end of the survey.]

SECTION A: BACKGROUND
Please answer the following questions about your background.

1. How do you best describe your agency/organization?
    a. Local Health Department
    b. Coalition
    c. Advocacy Group
    d. College or University
    e. K-12 School/School District
    f. Community-based Organization
    g. Voluntary Health Organization (e.g., American Diabetes Association, American Heart Association, etc.)
    h. Health Care Facility (e.g., hospital, clinic, medical health center)
    i. Government Agency
    j. For-profit business (non-health)
    k. Other (please specify)

2. Which of the following best describes your position?

    a. Top executive, health director, health officer, commissioner, or equivalent in the "office of the director"
    b. Administrator, deputy or assistant director
    c. Manager of a division or program
    d. Program coordinator
    e. Technical expert position (evaluator, epidemiologist, health educator)
    f. Other (please specify)

3. How long have you been in your current position?
   Years

4. Over your whole career how long have you been involved in public health?
   Years

5. Which degree/credentials do you hold (check all that apply)?
   Associate degree
   BS/BA
   CHES
   Certified in Public Health
   LPN
   RN
   BSN
   ARNP (Advanced Registered Nurse Practitioner)
   RD
   MS or MSc
   MPH or MSPH
   MSW
   MPA
   MA
   Other Masters Degree
   MD or DO
   PhD, DrPH, or ScD in a public health field
   PhD or ScD in another field
   EdD
   RS (Registered Sanitarian)
   Other (please specify below) _____________________

6. Are you…?
   Male
   Female
   Other
   Prefer not to answer

7. What is your race? (Check all that apply)
   White
   Black/African-American
   American Indian, Alaskan Native
   Asian
   Native Hawaiian or Other Pacific Islander
   Hispanic or Latino
   Other
   Prefer not to answer

8. What is your age?
   20-29 years
   30-39 years

40-49 years
50-59 years
60 years or older

9. Please indicate if you work in the below program areas by answering Yes or No.

a. Diabetes	Yes	No
b. Obesity	Yes	No
c. Physical Activity	Yes	No
d. Nutrition	Yes	No
e. Tobacco	Yes	No
f. Other (please specify):		

SECTION B: INTERVENTIONS TO ADDRESS CHRONIC DISEASES

You will read about various programs, policies and services intended to prevent or address chronic diseases. For each item, please indicate whether or not your agency has directly delivered each intervention, AND if your agency collaborated with community organizations to support the delivery of each intervention by responding "yes, no, or don't know." No single agency is expected to have implemented all the following interventions.

Hover your mouse over blue text for definition.

10. In the past year, has your agency directly delivered, and has your agency collaborated with organizations to support delivery of the following interventions?

	**Has your agency directly delivered/provided?**	**Has your agency collaborated with* organization(s) to support delivery?** [rollover definition: served as a community/clinical referral source, or a convener that facilitates the program and referral systems]
a. Diabetes prevention program/DPP - diet and physical activity promotion programs with people at increased risk for type 2 diabetes.	Yes No Don't know	Yes No Don't know
b. Community health workers* to deliver diet and physical activity promotion, and weight management to groups or individuals with increased risk for type 2 diabetes. [*Rollover definition: frontline, trained staff who are from or have a close understanding of the community served to bridge healthcare]	Yes No Don't know	Yes No Don't know
c. Diabetes self-management education with persons with diabetes delivered in community gathering places.	Yes No Don't know	Yes No Don't know
d. Diabetes management: identifying patients with diabetes and determining effective treatment.	Yes No Don't know	Yes No Don't know

e. Making physical activity easier through changes or new elements to transportation systems* *(sidewalks, trails, bicycle and public transit infrastructure)* AND land use* *(access to parks and rec facilities, increase in mix and closeness of where people live, work and play)*.	Yes No Don't know	Yes No Don't know
f. Programs or policies that increase physical activity in schools (e.g., time in school PE classes, training for PE teachers, equipment and materials, physical activity breaks).	Yes No Don't know	Yes No Don't know
g. Policies or changes that improve healthier food choices through nutrition assistance programs (e.g., WIC, SNAP, Senior Nutrition Programs).	Yes No Don't know	Yes No Don't know
h. Policies, environmental changes or programs promoting breastfeeding initiation, exclusive breastfeeding, and duration of breastfeeding.	Yes No Don't know	Yes No Don't know

SECTION C: YOUR VIEWS ON EVIDENCE-BASED INTERVENTIONS

This section asks about your views on **evidence-based interventions,** which are programs, policies, and services with evidence (based on published research) of improving health, such as those covered in the previous section. This refers to evidence-based strategies, like behavioral changes, changes to the physical environment, policy approaches, or clinical services that are specific and structured. Please indicate the extent to which you agree or disagree with the following statements.

11. Evidence-based interventions* are appropriate and relevant to the specific populations my agency serves.
[*Definition rollover: Policies, programs, and practices that are based on the best available public health science, knowledge, and data; refers to behavioral strategies, changes to the physical environment, policy approaches, or clinical services that specific and structured]

Strongly Disagree 1 2 3 4 5 6 7 Strongly Agree

12. I have the skills I need to adapt evidence-based interventions* from one setting or population to another.
[*Definition rollover – same as above]

Strongly Disagree 1 2 3 4 5 6 7 Strongly Agree

13. Program staff in my work group/division* (rollover definition) is aware of published evidence reviews of interventions (e.g., The Community Guide, Cochrane Reviews).

Strongly Disagree 1 2 3 4 5 6 7 Strongly Agree

14. Program staff in my work group/division is aware of toolkits for planning and evaluation.

Strongly Disagree 1 2 3 4 5 6 7 Strongly Agree

15. Do staff in your work group/division implement interventions to achieve health equity*?
[*Definition rollover - **health equity**: Ensuring all people have the opportunity to reach their highest level of health.]
Yes [If "Yes," go to Q20]
No [Skip to Q21]
Not Sure [Skip to Q21]

16. [If “Yes” to Q19] If possible, please share an example – an intervention your work group/division has implemented in the past 12 months to achieve health equity.
    Text response

SECTION D: EVIDENCE-BASED DECISION MAKING DEFINITIONS AND SUPPORTS

Now we are shifting away from specific interventions to a broader framework on use of an ***evidence-based process*** to plan and carry out public health programs, policies and practices in chronic disease control. Throughout the remainder of the survey, *such a process is referred to as* ***evidence-based decision-making*** and involves:

- Making decisions based on the best available scientific and/or rigorous program evaluation evidence;
- Applying program planning and quality improvement frameworks;
- Engaging the community in assessment and decision making;
- Adapting and implementing evidence-based interventions for specific populations or settings; and
- Conducting sound evaluation – the process of determining the relevance, effectiveness, and impact of activities according to their goals.

17. Within your agency, which of the following would most encourage you to utilize evidence-based decision-making* in your work? Using the list below, please rank the top three, where 1 is the most important. [Program Qualtrics for drag and drop]
    [*Prioritizing issues and implementing interventions based on sound science combined with community engagement, and evaluation.]

    Leaders in my agency placing a high priority on evidence-based decision-making
    Direct supervisors placing a high priority on evidence-based decision-making
    Positive feedback or encouragement to use evidence-based decision-making
    Easy access to data resources for evidence-based decision-making
    Support for travel to regional or national trainings or meetings *to learn about* evidence-based decision-making
    An employee performance evaluation that supports the use of evidence-based decision-making
    Professional recognition for use of evidence-based decision-making
    Making evidence-based decision-making part of job descriptions in my agency
    Support for travel to regional or national trainings or meetings *to present on* evidence-based decision-making
    Other: please specify

18. Which of the following would be most useful to you in building skills for evidence-based decision-making* in your work? From the items below, please rank the top three, where 1 is the most useful.

    General evidence-based decision-making training workshops
    Evidence-based decision-making training for specific program areas
    Access to experts by phone or email for questions about evidence-based decision-making
    Help with evidence-based decision-making processes (e.g. community assessment, evaluation)
    Evidence-based decision-making webinars
    Self-paced evidence-based decision-making internet programs or trainings
    Interactive evidence-based decision-making web-based meetings
    Learning to write summaries of research evidence (issue briefs)
    Learning to write policy briefs
    Network of health departments and agencies that meets online
    Network of health departments and agencies that meets in person
    Other: please specify

Adoption and Implementation of evidence to Mobilize Local Health (AIM-Local Health)
Quantitative Survey

SECTION E: IMPORTANCE AND AVAILABILITY OF EVIDENCE-BASED DECISION MAKING

Now, we would appreciate your help rating the importance and availability of each skill in the statements below. First, read the statements (skills in evidence-based decision-making) below; then, use the first scale to rate the importance of each of the skills to you. Next, use the second scale to rate how available each skill is to you when you need it (either in your own skill set or among others in your agency).

19. Community assessment: Understand how to describe the health issue according to the needs and assets of the population/community of interest.

Unimportant	0	1	2	3	4	5	6	7	8	9	10	Very Important
Not Available	0	1	2	3	4	5	6	7	8	9	10	Very Available

20. Quantifying the issue: Understand the uses of descriptive epidemiology (i.e., compiling and analyzing data by person, place, and time) in quantifying a public health issue.

Unimportant	0	1	2	3	4	5	6	7	8	9	10	Very Important
Not Available	0	1	2	3	4	5	6	7	8	9	10	Very Available

21. Prioritization: Understand how to prioritize program and policy options.

Unimportant	0	1	2	3	4	5	6	7	8	9	10	Very Important
Not Available	0	1	2	3	4	5	6	7	8	9	10	Very Available

22. Action planning: Understand the importance of developing an action plan for how to achieve goals and objectives.

Unimportant	0	1	2	3	4	5	6	7	8	9	10	Very Important
Not Available	0	1	2	3	4	5	6	7	8	9	10	Very Available

23. Adapting interventions: Understand how to modify programs and policies for different communities and settings.

Unimportant	0	1	2	3	4	5	6	7	8	9	10	Very Important
Not Available	0	1	2	3	4	5	6	7	8	9	10	Very Available

24. Evaluation designs: Understand the different designs that are useful in program or policy evaluation (i.e., quasi-experimental designs).

Unimportant	0	1	2	3	4	5	6	7	8	9	10	Very Important
Not Available	0	1	2	3	4	5	6	7	8	9	10	Very Available

25. Quantitative evaluation: Understand the uses of quantitative evaluation approaches (e.g., surveillance and/or surveys).

Unimportant	0	1	2	3	4	5	6	7	8	9	10	Very Important
Not Available	0	1	2	3	4	5	6	7	8	9	10	Very Available

26. Qualitative evaluation: Understand the value of qualitative evaluation approaches (e.g., focus groups, key informant interviews) including the steps involved in conducting qualitative evaluations.

Unimportant	0	1	2	3	4	5	6	7	8	9	10	Very Important

Not Available	0	1	2	3	4	5	6	7	8	9	10	Very Available

27. Economic evaluation: Understand how to use economic data (e.g., cost-effectiveness) in the decision making process.

Unimportant	0	1	2	3	4	5	6	7	8	9	10	Very Important
Not Available	0	1	2	3	4	5	6	7	8	9	10	Very Available

28. Communicating evidence to decision-makers: Understand the importance of effectively communicating with decision-makers about public health issues (e.g., elected officials, superintendents, business leaders, or other key community partners).

Unimportant	0	1	2	3	4	5	6	7	8	9	10	Very Important
Not Available	0	1	2	3	4	5	6	7	8	9	10	Very Available

SECTION F: SPREADING EVIDENCE-BASED DECISION MAKING
The next sections of the survey will help us understand readiness to support use of evidence-based decision-making*. (*EBDM rollover) For the following statements, please indicate the extent to which you agree or disagree. Please remember that your responses are anonymous and only summary findings that do not identify people or organizations will be reported.
*[Definition rollover – evidence-based decision-making: Prioritizing issues and implementing interventions based on sound science combined with community engagement, sound management, and evaluation.]

Hover your mouse over blue text for definition.

[Note the following denotes final factor items per confirmatory factor analysis:
Mazzucca S, Parks RG, Tabak RG, Allen P, Dobbins M, Stamatakis KA, Brownson RC. Assessing Organizational Supports for Evidence-Based Decision Making in Local Public Health Departments in the United States: Development and Psychometric Properties of a New Measure. J Public Health Manag Pract. 2019 Sep/Oct;25(5):454-463. doi: 10.1097/PHH.0000000000000952. PMID: 31348160; PMCID: PMC6614014.]

Factor 1: Awareness of culture supportive of EBDM (3 items).

Factor 2: Capacity and expectations for EBDM (7 items).

Factor 3: Resource availability (3 items).

Factor 4: Evaluation Capacity (3 items).

Factor 5: EBDM climate cultivation (3 items).

Factor 6: Partnerships to Support EBDM (3 items).

Adoption and Implementation of evidence to Mobilize Local Health (AIM-Local Health)
Quantitative Survey

Awareness

F1 29. I am provided the time to identify evidence-based programs and practices.

Strongly Disagree 1 2 3 4 5 6 7 Strongly Agree

F1 30. My direct supervisor recognizes the value of management practices* (provide rollover) that facilitate evidence-based decision-making.
[*For example, asking for employee input, hiring well-trained staff, supporting learning, building partnerships, and using effective budget practices.]

Strongly Disagree 1 2 3 4 5 6 7 Strongly Agree

F1 31. My work group/division* offers employees opportunities to attend evidence-based-decision making trainings. [rollover work group/division definition]

Strongly Disagree 1 2 3 4 5 6 7 Strongly Agree

32. Top leadership in my agency (e.g., director, assistant directors) recognizes the value of evidence-based decision-making.

Strongly Disagree 1 2 3 4 5 6 7 Strongly Agree

Use of EBDM

F2 33. I use evidence-based decision making in my work.

Strongly Disagree 1 2 3 4 5 6 7 Strongly Agree

F2 34. My direct supervisor expects me to use evidence-based decision making.

Strongly Disagree 1 2 3 4 5 6 7 Strongly Agree

F2 35. My performance is partially evaluated on how well I use evidence-based decision making in my work.

Strongly Disagree 1 2 3 4 5 6 7 Strongly Agree

F2 36. My work group/division* currently has the resources (e.g. staff, facilities, partners) to support application of evidence-based decision making.

Strongly Disagree 1 2 3 4 5 6 7 Strongly Agree

F2 37. The staff in my work group/division* has the necessary skills to carry out evidence-based decision making.

Strongly Disagree 1 2 3 4 5 6 7 Strongly Agree

F2 38. The majority of my work group/division's* external partners support use of evidence-based decision making.

Strongly Disagree 1 2 3 4 5 6 7 Strongly Agree

F2 39. Top leadership in my agency encourages use of evidence-based decision making.

Strongly Disagree 1 2 3 4 5 6 7 Strongly Agree

Resource Maintenance

F3 40. Informational resources (e.g. academic journals, guidelines, and toolkits) are available to my work group/division* to promote the use of evidence-based decision making.

Strongly Disagree 1 2 3 4 5 6 7 Strongly Agree

F3 41. My work group/division* engages a diverse external network of partners that share resources* to facilitate evidence-based decision making. [rollover resources for EBDM examples: For example, staff, facilities, data, toolkits, journal articles, access to training]

Strongly Disagree 1 2 3 4 5 6 7 Strongly Agree

F3 42. Stable funding is available for evidence-based decision making.

Strongly Disagree 1 2 3 4 5 6 7 Strongly Agree

Evaluation Maintenance

43. My work group/division* supports community needs assessments to ensure that evidence-based decision making approaches continue to meet community needs.

Strongly Disagree 1 2 3 4 5 6 7 Strongly Agree

F4 44. My work group/division plans for evaluation of interventions prior to implementation.

Strongly Disagree 1 2 3 4 5 6 7 Strongly Agree

F4 45. My work group/division uses evaluation data to monitor and improve interventions.

Strongly Disagree 1 2 3 4 5 6 7 Strongly Agree

F4 46. My work group/division distributes intervention evaluation findings to other organizations that can use our findings.

Strongly Disagree 1 2 3 4 5 6 7 Strongly Agree

Organizational Climate

47. My work group/division* has access to evidence-based decision making information that is relevant to community needs.

Strongly Disagree 1 2 3 4 5 6 7 Strongly Agree

48. When decisions are made within my work group/division*, program staff members are asked for input.

Strongly Disagree 1 2 3 4 5 6 7 Strongly Agree

F5 49. Information is widely shared in my work group/division* so that everyone who makes decisions has access to all available knowledge.

Strongly Disagree 1 2 3 4 5 6 7 Strongly Agree

F5 50. My agency is committed to hiring people with relevant training or experience in public health core disciplines (e.g. epidemiology, health education, environmental health).

Strongly Disagree 1 2 3 4 5 6 7 Strongly Agree

F5 51. My agency has a culture that supports the processes necessary for evidence-based decision making.

Strongly Disagree 1 2 3 4 5 6 7 Strongly Agree

Relationships and Partnerships

52. Our collaborative partnerships have missions that align with my agency.

Strongly Disagree 1 2 3 4 5 6 7 Strongly Agree

F6 53. It is important to my agency to have partners who share resources (money, staff time, space, materials).

Strongly Disagree 1 2 3 4 5 6 7 Strongly Agree

F6 54. It is important to my agency to have partners in healthcare to address population health issues.

Strongly Disagree 1 2 3 4 5 6 7 Strongly Agree

F6 55. It is important to my agency to have partners in other sectors (outside of health) to address population health issues.

Strongly Disagree 1 2 3 4 5 6 7 Strongly Agree

SECTION H: YOUR WORK STYLE

This next section asks about your work style and how passionate and persevering you see yourself to be. Below are a number of statements that may or may not apply to you. There are no right or wrong answers, so just answer honestly, considering how you compare to most people. Please remember your responses are anonymous and only summary findings that do not identify people or organizations will be reported.

	Not Like Me At All (1)	Not Much Like Me (2)	Somewhat Like Me (3)	Mostly Like Me (4)	Very Much Like Me (5)
New ideas and projects sometimes	❍	❍	❍	❍	❍

distract me from previous ones. (1)					
Setbacks don't discourage me. I don't give up easily. (2)	❍	❍	❍	❍	❍
I have been strongly focused on a certain idea or project for a short time but later lost interest. (3)	❍	❍	❍	❍	❍
I am a hard worker. (4)	❍	❍	❍	❍	❍
I often set a goal but later choose to pursue a different one. (5)	❍	❍	❍	❍	❍
I have difficulty maintaining my focus on projects that take more than a few months to complete. (6)	❍	❍	❍	❍	❍
I finish whatever I begin. (7)	❍	❍	❍	❍	❍
I am diligent. (8)	❍	❍	❍	❍	❍

THANK YOU!

Thank you for completing this survey! Your time, efforts, and responses are greatly appreciated by all of us at the Prevention Research Center in St. Louis. Your efforts today will help us plan how best to support local health agencies as we move forward and assess strategies to support evidence-based efforts.

If you have questions, please call Renee Park.

To accept your optional $20 Amazon gift card, please go to the following link: [place link here]

> *It is a separate website where you will be asked to give your e-mail address. If you choose to accept the gift card you will also be asked personal information so that Washington University in St. Louis can process and send your gift card to you. The information cannot be traced to your survey responses and will be kept confidential.*
