# Supplementary material for: How to “Start Small and Just Keep Moving Forward”: Mixed Methods Results From a Stepped-Wedge Trial to Support Evidence-Based Processes in Local Health Departments
Source: Front Public Health. 2022 Apr 28;10:853791. doi: 10.3389/fpubh.2022.853791 (PMC9096224; doi:10.3389/fpubh.2022.853791)
Supplement: Supplementary Material 3 — Qualitative interview guide. These questions and prompts were used in interviews with local health department staff to guide a discussion around facilitators and barriers to implementing evidence-based processes within their respective agencies. [file Data_Sheet_2.pdf]

AIM–Local Health Case Study Interview Guide

<u>Introduction</u>

Hello. My name is _______________. I am with the Prevention Research Center at Washington University in St. Louis and I am calling regarding a project to understand how local health departments (LHDs) have been supporting and using evidence-based processes since participation in the AIM-Local Health project.

Thank you for agreeing to do this interview. Is this still a good time to talk?

[YES: continue reading below]

[No: Is there a better time we could schedule this interview for to best fit your schedule?]

We want to learn your views on what makes it easier or harder to get supports into place to prepare and support staff in planning, carrying out and evaluating evidence-based work. Your responses are confidential, so please speak frankly. No one's feelings will be hurt, and speaking candidly will help the project and future work.

For the purposes of this interview, we define evidence-based decision-making (EBDM)/evidence-based <u>processes</u> as those using the following approaches:

- Making decisions based on the best available scientific evidence;
- Applying program planning and quality improvement frameworks (including community health assessment methods and prioritization methods);
- Engaging the community and partners who work with communities in assessment and decision making;
- Adapting and implementing evidence-based interventions for specific populations or settings; and
- Conducting sound evaluation.

This interview will take about 40 minutes. With your permission, we would like to audio record our conversation for note-taking purposes. As I mentioned, your responses are confidential, and will not be used in any way to identify you or your health department. You will not be identified in any write-ups and no one will know who said what.
We will gladly share what we learn with you.
You also have the option to receive a $30 Amazon gift card in appreciation of your time.
You may skip any questions you do not feel comfortable responding to, and you are able to stop the interview at any time.

Would you like to proceed with this interview?

[YES: Thank you. I will be turning the device on now. **Continue reading below]**

[NO: Thank you for your time and consideration about this project. We will not contact you further about participating in these interviews**. END THE PHONE CALL].**

Do you have any questions before we get started?

<u>Part 1: EBDM Process – Capacity-building supports</u>

1.1 Since [Mar/Apr 2018 | Nov/Dec 2018 | July/Aug 2019], what – if anything – has been added or changed with policies and practices to support use of EBDM/evidence-based processes **<u>within your work group/division* or your health department overall</u>**?

*Work group/division: The grouping of programs in the same administrative area where you work (health promotion, community health, chronic disease or other).

Possible prompts:

- What management practices are in place to support use of EBDM/evidence-based practice? Which of these have been set up since [Mar/Apr 2018 | Nov/Dec 2018 | July/Aug 2019]?
- What has your work group/division or department overall done to encourage your staff to use EBDM?
- How does your work group/division or department overall ensure staff are prepared to use EBDM?
- What has your work group/division or department overall done to prepare your staff to use EBDM?
    - For workforce development in EBDM? Any orientations or trainings in EBDM since [Mar/Apr 2018 | Nov/Dec 2018 | July/Aug 2019]?
    - For accreditation preparations?
    - Any all staff meetings to communicate about EBDM/evidence-based practice across work groups/divisions?

1.2 How would you describe your work group/division and the overall health department environment as it relates to using evidence-based processes? Some people call this organizational climate and culture.

Possible prompts if needed:

- How have the management practices to support EBDM been received?
- To what extent has use of EBDM/evidence-based practice been accepted?

[Now I would like to find out about support for EBDM processes] Part 2: EBDM Process Support – Feedback, Facilitators, Barriers [10 mins]

Thinking of the management practices and other evidence-based processes you described at the beginning…which one(s) were the most useful or successful? [Pause – let them tell you]

The next questions will ask about this/these element(s).

2.1 How well or poorly has X been accepted/used?

Prompt: What feedback have you received or heard about X?

2.2 What difference has X made in the day-to-day work of you and your colleagues?

2.3 What has made it easier to get X management practice in place to support EBDM use?

Possible prompts:

- What has been instrumental in promoting the use of EBDM?
- What factors have contributed to the use of EBDM?

2.4 Were there challenges encountered, and if so, how were challenges addressed?

Possible prompts:
What has made it hard to get X management practice into place to support EBDM use?

[If there's time, ask Q2.1-2.4 for a second successful/useful EBDM process support.]

[So, now I'm going to ask you a little bit about partners]

Part 3: Partners

3.1 Have any supports or expectations been created for partnering organizations for evidence-based processes since [Mar/Apr 2018 | Nov/Dec 2018 | July/Aug 2019]? If yes, what are they?

Prompts for LHD staff:

- What changes, if any, have you detected in the ways your work group/division or department overall interacts with or supports partners in evidenced-based processes?
- What changes, if any, have you noticed in the ways health department staff interact with partners regarding use of evidence-based program planning and evaluation, or use of evidence-based strategies?

3.2 What changes in procedures or management practices, if any, have you noticed partners making to support EBDM in their agencies?

[I'm going to transition to questions about sustaining EBDM processes and recommendations you may for other LHDs]

Part 4: Sustaining EBDM Processes and Recommendations for Other LHDs

4.1 How do staff in your department view EBDM amidst the additional demands of COVID19/the pandemic?

4.2 At the beginning of the interview, we discussed management practices your health department put in place. How, if at all, have the additional demands during COVID19/the pandemic affected the procedures and management practices you described?

Possible prompts:

- Have the changes you described persisted through the pandemic?
- What, if any, steps has your department taken to help sustain the EBDM support procedures and management practices through COVID19/the pandemic?

4.3 Given the demands of COVID19 on your health department—and thinking about the future if a university/academic unit approaches you to collaborate on a research project, what things could a university/academic partner do or offer that may make a research collaboration more helpful and productive?

4.4 What steps, if any, have been taken to sustain the use of EBPH/EBDM in your work group/division or agency overall that have not already been discussed?

Prompts:

- What has your work group/division or agency overall done to institutionalize EBDM use?
- What structures or management practices has your work group/division or agency overall put in place to ensure current and future staff are prepared to use evidence-based processes?

4.5 Are there any other steps you would like to see put into place in your work group/division or agency overall to reinforce these practices that we have not already discussed?

Prompt:

- What management practices would you like to see in the future to support capacity for EBDM

4.6 What additional resources do you need – or would be helpful to support and expand the implementation of EBDM processes – division/work group or organization?

Possible prompts only if needed: access to professional journals/articles, dedicated program evaluation staff, data analyst

4.6 What advice do you have for other health departments like yours that want to build additional capacity for and use of EBDM?

[We're nearing the end of the interview…and now I'd like to get feedback on the project]

Part 5: Project Feedback:

5.1 What do you think worked well?

5.2 We've been working with you for [time period: 2 years | almost 1 ½ years | about 8 months]. If we were to start this with other local health departments, what would you recommend we do differently?

Closing Question: Is there anything else you'd like to share that you haven't already?

Part 6: Demographics

Thank you. Before we close, we have a few quick questions about your background that we'll only use in summary form across people interviewed, to summarize in our reports.

6.1 [LHD/Partner status: record whether the person is a LHD employee or partner.]

6.2 How long have you been in your current position?

6.3 How long have you been with this agency or organization?

6.4 How long have you worked in public health overall?

Closing

Thank you very much for taking the time to talk with us about this study.

In appreciation for your time, you have the option to receive a $30 Amazon gift card. Would you like to receive the gift card?

> **[YES**: we will send you a 'thank you' email that will include a link to a brief survey that will collect the needed information for us to send you a $30 Amazon gift card by email.**]**
>
> **[NO: continue reading below]**

Thank you, again for your time and perspective. If you have any questions, contact Renee Parks, project manager. Have a great rest of your day!
